# Design, Simulation and Functional Testing of a Novel Ankle Exoskeleton with 3DOFs

**DOI:** 10.3390/s24196160

**Published:** 2024-09-24

**Authors:** Gani Sergazin, Nursultan Zhetenbayev, Gulzhamal Tursunbayeva, Arman Uzbekbayev, Aizada Sarina, Yerkebulan Nurgizat, Arailym Nussibaliyeva

**Affiliations:** 1 Global Education & Training, University of Illinois at Urbana-Champaign, Champaign, IL 61820, USA; 2 Department of Information Security, Eurasian National University, Astana 10000, Kazakhstan; 3 LARM2: Laboratory of Robot Mechatronics, University of Rome Tor Vergata, 00173 Rome, Italy; 4 Department of Electronics and Robotics, Almaty University of Power Engineering and Telecommunications, Almaty 050013, Kazakhstan; 5 Research Institute of Applied Science and Technologies, Almaty 050013, Kazakhstan

**Keywords:** design and simulation, biomechanics, ankle exoskeleton, motion assisting devices

## Abstract

This paper presents a study on developing a new exoskeleton for ankle joint rehabilitation with three degrees of freedom (3 DOFs). The primary attention is paid to the process of designing and modelling the device aimed at restoring the lost functions of joint mobility. The authors conducted a complex analysis of the functional requirements of the exoskeleton based on research into the potential user’s needs, which allowed for the development of a conceptual model of the proposed device. In this study, a prototype of the exoskeleton is designed using modern additive technologies. The prototype underwent virtual testing in conditions maximally close to reality, which confirmed its effectiveness and comfort of use. The main results of this study indicate the promising potential of the proposed solution for application in rehabilitation practices, especially for patients with ankle joint injuries and diseases.

## 1. Introduction

The musculoskeletal system includes bones, joints, ligaments, muscles, and their associated nerve formations. This combination forms a single system, that provides mechanical protection for a person’s internal organs and performs support tasks, enabling movement. Disruptions or damage to any part of this system can have serious negative consequences. These include more than 150 different types of diseases and conditions, such as injuries to bones, fractures, and joint injuries, including sprains or tears of ligaments and muscles, as well as damage to nerve bundles, resulting in temporary or lifelong limitations in function and movement in everyday life [1]. In this case, rehabilitation and medical care play a crucial role in restoring musculoskeletal function following injuries and chronic diseases. These interventions are essential for regaining and improving mobility and overall quality of life.

As an essential health service, rehabilitation plays a crucial role in achieving universal health coverage in any country. According to the World Health Organization, approximately 2.4 billion people worldwide are living with health problems that can be improved through rehabilitation [2]. Of these, about 1.71 billion people suffer from musculoskeletal disorders, among whom 86 million require rehabilitation processes following a stroke. Given the changes occurring in the health status and characteristics of the population worldwide, the estimated need for rehabilitation will only increase in the coming years [3], which emphasizes the importance of research into the development and implementation of the latest rehabilitation technologies and techniques to improve the quality of life and health of the population.

One of the key areas of development in this field is the creation of a wide range of devices used in medical practice in rehabilitation, from simple instruments to complex life support systems and surgical robots. These innovations help to combat various musculoskeletal conditions, which ultimately improves diagnosis and treatment processes, and accelerates the recovery process, which facilitates more efficient interaction between patients and doctors.

The impact of such devices on improving the quality of life and health of the population is invaluable. They are widely used in various fields of medicine and health care, such as orthopedics and traumatology, neurology and neurorehabilitation, physiotherapy, and sports medicine, as well as cardiology and respiratory therapy. These devices have many advantages. They help accelerate recovery processes, improve the functional capabilities of the body, reduce the risk of complications after illness or injury, and increase patients’ motivation for rehabilitation [4]. In addition, these devices allow the development of individualized recovery programs tailored to the characteristics and needs of each specific patient. This contributes to the best results and effective treatment, helping patients return to a full life and become more independent and active in their daily activities.

Despite these advantages, rehabilitation of the musculoskeletal system currently faces many challenges due to the cost of developing technology, lack of resources, including the lack of assistive technology, equipment, and devices, as well as consumables, and the need for additional research and data collection on the rehabilitation process [5]. Given the importance and significance of the rehabilitation process in patients today, in most cases, the rehabilitation process is carried out mainly manually by physicians. This approach faces problems such as high labor intensity, dependence on physician experience, difficulty in accurately controlling exercise parameters and evaluating the effects of rehabilitation, which raises the urgency for research in the creation of accessible modern technologies, automated rehabilitation systems, and devices that provide more accurate control of exercise parameters and allow the collection of data on their effectiveness, which can lead to more personalized treatment plans.

Introducing such innovations into rehabilitation requires a comprehensive approach, including training specialists, integrating new technologies into clinical practice, and continuously monitoring results. This will ultimately reduce the risk of errors and increase patients’ motivation to quickly return to a full life. This process can be an essential step towards more effective and affordable rehabilitation methods and offering patients increasingly effective and convenient solutions.

The development and production of rehabilitation devices is a complex and multi-stage process involving many aspects, starting with research, design, and prototype development, followed by virtual and practical testing to identify possible problems and deficiencies. Compliance with all standards and regulations set in the medical device field is also an essential part of the process. In addition, it is necessary to consider the specifics of using the device for medical purposes, such as the possibility of prolonged contact with the human body, to maximize the safety and reliability of the device [6]. Among such devices, robotic exoskeletons have been widely used to help patients after strokes, brain injuries, spinal cord injuries, and for patients with muscle or joint diseases. They are also effective in rehabilitating patients with various ankle injuries. These devices aim to improve movement coordination, strengthen muscles, increase joint mobility, and improve balance [7].

Due to the high activity of daily life, the ankle joint becomes one of the most vulnerable joints in the human musculoskeletal system, and it is characterized by a complex structure, significant load, and flexibility of movement, playing an integral role in daily life. In addition, diseases such as stroke and spinal cord injuries can cause muscle weakness, spasticity, poor control of ankle joint movements, or even deformity [8], which highlights the significance of research in the field of creating devices for human lower extremity rehabilitation.

Today, researchers worldwide are developing various types of rehabilitation devices using a wide range of methods and solutions, which raises the demand for creating new, innovative, and affordable types of rehabilitation devices. Exoskeletons are emerging as an innovative lower limb solution to improve function for people with lower limb injuries. These devices are vital in restoring lost function after musculoskeletal diseases and injuries. Therefore, this study discusses the development of a new exoskeleton for ankle rehabilitation from a design and modeling perspective. This development will be capable of restoring the lost mobility functions of the ankle joint. The process of creating accessible exoskeletons for ankle rehabilitation represents a significant step forward in biomedical engineering and involves several key aspects, as reviewed by the authors in this study. The first phase of this research to determine the basic functional requirements for an exoskeleton, we first conducted a thorough investigation of the needs of potential users, including an analysis of their lifestyle, types of ankle injuries and diseases, and the impact of such devices on the current health status of the rehabilitated patient in a human-machine interaction environment. The next stage involves conceptual design, where the initial concept of the exoskeleton is developed. This includes selecting key components such as power transmission mechanisms, control systems, and sensors to monitor joint position and load. It is also important to consider the device’s aesthetics and usability during rehabilitation. The final stage involves developing and virtual testing an ankle exoskeleton prototype. The prototype is developed and tested virtually in conditions as close to real life as possible to assess its effectiveness, reliability, and comfort of use. After conducting all the above stages, the we created an accessible, inexpensive exoskeleton prototype with three degrees of freedom, which will become an effective means of rehabilitation for patients with various injuries and traumas of the ankle joint. The widespread use of such devices in clinical practice opens vast opportunities for restoring patient motor function. They allow for improvement in the quality of life of rehabilitated patients, thus increasing their level of independence and mobility and making the rehabilitation process more effective and personalized.

The main feature of the proposed rehabilitation exoskeleton is the presence of three degrees of freedom, which allows for the effective control of the movement of the ankle joint in all its possible states. This enables the device to mimic the natural movements of the ankle joint, creating a smoother and more natural walking experience for the patient, which speeds up the rehabilitation process. Such an exoskeleton may be helpful to patients in chronic disease management processes and rehabilitation following ankle injuries such as fractures or ligament changes. This device can be applied to support people with difficulty walking due to age-related changes in their joints.

## 2. Related Work

The inability to walk due to conditions such as stroke, musculoskeletal disorders, and spinal cord injuries is a serious concern today. In many cases, injuries of this nature cause loss of motor function in the lower extremities, encompassing the hip, knee, ankle, foot, or a combination of these. In such situations, restoring lost or limited mobility of the lower limbs using robotic rehabilitation significantly improves the patient’s quality of life, allowing them to become independent and active again [9,10]. Over the last decade, robotic rehabilitation of the lower limbs has significantly improved. Today, it is a high-tech solution that helps restore the motor and functional capabilities of patients with movement disorders. In this case, the effectiveness of these solutions is assessed by a comprehensive approach and close interaction between the rehabilitation device, patient, physician, physiotherapist, and other specialists. More affordable and effective ways to improve rehabilitation include using robotic exoskeletons to restore lower limb function [11]. In this direction, researchers worldwide are developing new technologies to improve human lifestyles using different approaches and development methods. Many modern rehabilitation exoskeletons have been developed, and mechanisms, control systems, sensors, etc., have been improved. All these studies can be a strong background for developing a new rehabilitation robotic exoskeleton, which is presented in this study.

Many studies in the field of musculoskeletal rehabilitation devices have focused on the development and testing of exoskeletons to restore ankle joint function. Much of the research and development has focused on the rehabilitation of patients after stroke, as stroke can lead to severe motor impairment and loss of function, and in the worst case, death [12]. The consequences of stroke can lead to partial or total disability, which affects a person’s muscle strength and functional mobility in general. In countries around the world, stroke remains a common public health problem and is the second leading cause of human mortality, with an annual incidence of about 180 cases per 100,000 people [13]. According to the source [14], after stroke, one-third of survivors live longer than three months, and a significant proportion of ambulatory patients lose upper and lower limb mobility functions. Subsequently, they must use wheelchairs and other assistive devices, experiencing a marked decrease in walking speed and endurance. Consequently, restoring and optimizing ambulatory function after stroke is paramount in facilitating social and vocational reintegration. Among stroke patients, the introduction of exoskeleton technology is promising, given the increasing prevalence of stroke and associated mortality rates. In this regard, the authors of the research [15] emphasize the importance of considering the biomechanical effects of the exoskeleton to enhance rehabilitation efficiency and patient comfort. Experiments were conducted using AGoRA and T-FLEX exoskeletons (WE&T). The results of the study show a significant reduction in muscle activity by 50% in the biceps femoris, 59% in the lateral calf muscle, and 35% in the tibialis anterior muscle when using the T-FLEX and AGoRA exoskeletons together. The use of this development in clinical practice can significantly reduce muscle activity without changing gait or stability parameters but cannot significantly affect the overall gait. The authors [16] demonstrated a prototype with a five-link mechanical system having two degrees of freedom (2DOF). The key innovation is the anthropomorphic structure of the exoskeleton. It includes a variable instantaneous center of rotation (ICR) that can adapt to the individual knee joint variability. However, angular coordination of exoskeleton movements can be disturbed due to inconsistent synchronization of movements, which requires additional re-adjustment.

Other applications of rehabilitation exoskeletons have been studied by authors from different countries, including their use as an aid for people with spinal cord injuries (SCI) characterized by paralysis [17,18]. According to global estimates by the World Health Organization, about 15 million people are living with spinal cord injuries worldwide. Without effective prevention, treatment and rehabilitation can progress to severe and even life-threatening secondary conditions that lead to premature death of the individual [19]. Interventions in the acute phase prioritize non-emergency medical care, while the chronic phase entails persistent complications that require long-term rehabilitation and nursing support. Moreover, spinal cord injuries (SCI) often result in paralysis of the lower extremities, leading to complete loss of motor function in these limbs. In such situations, the application of exoskeletons in spinal cord injury rehabilitation shows significant potential and is becoming increasingly in demand due to its unique advantages. In this direction, the authors [20,21,22] discuss current trends in exoskeleton design and their integration with modern technologies such as artificial intelligence (AI), augmented (AR) and virtual reality (VR), and the Internet of Things (IoT). The listed studies emphasize the importance of developing lightweight, flexible, and intelligent exoskeletons capable of integration into everyday activities, which is highly relevant today. Despite the generalization of the listed studies, the authors did not fully consider the specific needs and physiological features of each case of exoskeleton application in the rehabilitation of spinal cord injury.

High biomechanical stresses in the workplace can also lead to a high risk of occupational musculoskeletal disorders. This creates the need for various assistive devices. Today, the scope of rehabilitation exoskeletons is expanding, and they are also being used in industries to reduce the strain on workers and provide prevention of various types of injuries. They can provide additional energy for human movement, which can be used in multiple fields, such as military, industrial, and medical rehabilitation [23]. In this direction, the authors of the study [24,25,26,27,28] have developed industrial and vocational exoskeleton robots that provide physical assistance to workers in industrial facilities, enhanced by the use of various bio-signal measurement sensors and artificial intelligence.

Rehabilitation exoskeletons also play a vital role in the recovery process from various joint injuries by providing additional support and mobility, which helps prevent further deterioration of the joints and ligaments of the musculoskeletal system. Compared to other parts of the lower extremities, the ankle joint is subjected to significant stresses and strains, which makes it particularly vulnerable to various types of injuries, such as sprains and fractures [28]. In addition, the authors of the study [29] believe it is the most commonly injured joint in more than 70 sports, including aerobic exercise, rock climbing, indoor volleyball, mountaineering, basketball, track and field, and others. Other authors [30] state the negative impact of ankle injuries on everyday activities, further reducing productivity and exerting social and economic pressures. These injuries impair quality of life by causing symptoms such as pain, swelling, limited mobility, weakness, and reduced joint function.

Without adequate treatment and rehabilitation, the aforementioned musculoskeletal disorders of the human lower extremities can lead to fractures, functional instability, decreased muscle strength, impaired proprioception, restricted activity, disability, and permanent hypodynamia. There are many treatments for musculoskeletal disorders, such as physiotherapy, massage and manual therapy, therapeutic exercises (PT), mechanotherapy, and others. These techniques have attracted considerable public attention over the past decade and are constantly being improved. One of the most effective approaches to date is the use of robotic exoskeletons.

Robotic exoskeletons are external apparatuses affixed to the human body, designed to enhance physical abilities beyond one’s innate capabilities. Rehabilitation exercises are generally categorized as passive (P) or active (A) modalities. Passive exercises involve therapists or robotic systems assisting subjects in moving affected body parts, while active exercises require independent effort from the subject. As patient loads increase, therapists may experience heightened levels of stress, potentially diminishing the effectiveness of rehabilitation and patient engagement. This, consequently, can impede functional independence and the ability to perform everyday activities. Incorporating exoskeletal rehabilitation devices offers promising solutions to these issues, significantly improving the effectiveness of rehabilitation. Numerous studies have highlighted the relationship between the restoration or improvement of motor skills and the performance of demanding, repetitive functional tasks [17].

Despite significant progress in the field of rehabilitation robotics, there are still open problems in achieving the main goal of restoring body functions. Challenges persist, including limitations in hardware systems and control mechanisms such as restricted range of motion, complexity, high cost, discomfort, excessive weight, incompatibility parameters with human anatomy, lack of safety features, and inefficient power transmission methods. These factors collectively contribute to the hurdles faced in the advancement of rehabilitation robotics.

Recognizing the critical role of exoskeletal robotics in modern medical rehabilitation in recent years, we have witnessed intensive studies elucidating the characteristics and drawbacks of these devices. Studies have compared prices, weight, efficiency, power sources, and control systems [31]. In contrast, others have focused on materials, actuator mechanisms, manufacturing techniques [32], and emerging trends in 3D-printed lower limb exoskeletons [33]. Other studies have delved into control strategies for lower limb exoskeletons [34]. In addition, many contributions have focused on predicting and understanding individual responses to rehabilitation devices. In [35], researchers tested which data could be used for accurate characterization. It was found that mismatch modeling can improve the understanding of ankle exoskeleton responses by analyzing changes in motion.

The development of robotics in healthcare, from surgical applications in the 1960s to rehabilitation in the 1990s, has resulted in various available technologies with different control systems and levels of freedom. This is supported by the research of [35], which provides an in-depth analysis of the use of robotics in healthcare. Dong et al. [36] introduced a novel ankle rehabilitation system with three training strategies based on a hospitalization controller. It consisted of developing a robotic exoskeleton for different levels of muscle strength and stages of rehabilitation. This robot for parallel ankle rehabilitation has three rotational degrees of freedom, two linear servos, and a servo motor. It is mentioned that new 3D printing materials and methods are still in the pilot and research stages, and their application in clinical practice requires further standardization and scalability. The latest materials and methods mentioned by the authors are still in the research stage, and require further validation and application in clinical practice. In such studies, the authors [37] presented a novel hybrid ankle rehabilitation mechanism capable of adapting to different lower extremity sizes in adults. This mechanism includes three modes of ankle joint motion without axis displacement and incorporates backward and forward position/kinematics analysis using closed-loop vector methods and particle swarm optimization algorithms. Numerous studies have also investigated robotic devices’ significant benefits for patients [38,39,40,41]. Although the abilities of exoskeletons can perform tasks such as data collection, report generation, and evaluation of patient progress, they require trained personnel, and incur high costs, as cited in [42].

Access to adequate and convenient rehabilitation devices is crucial for improving the quality of life of patients. Innovations in and the development of such devices can significantly enhance the accessibility and effectiveness of rehabilitation, making rehabilitation more available and user-friendly for all patients. This highlights the urgency of developing innovative rehabilitation devices that are compact, lightweight, and easy to use, with straightforward control system configurations. The cost of rehabilitation robotic devices and their maintenance remains a critical factor limiting the widespread adoption. Therefore, developing efficient and cost-effective solutions is a significant challenge that has the potential to greatly improve the convenience, accessibility, and overall effectiveness of rehabilitation.

In this study, we present a new exoskeleton solution designed specifically for the ankle joint. The mechanism utilizes electric linear actuators to facilitate user movements and enhance mobility. The principal innovation of the study is the presence of three degrees of freedom, which promote the recovery of the ankle joint and foot. In the exoskeleton structure, each degree of freedom serves a distinct function. The first degree of freedom pertains to the vertical movement of the leg, allowing it to be raised and lowered. The second degree of freedom involves rotation around the ankle axis, enabling the foot to rotate in and out. The third degree of freedom provides upward and downward movement relative to the heel, which is crucial for everyday walking and balance. It is hypothesized that the exoskeleton solution proposed by the authors will ensure its suitability for patients across various demographic groups and will be affordable. The device’s performance was evaluated and analyzed through virtual functional testing, including range of motion assessments performed independently and in conjunction with normal subjects.

Comprising five sections, this study offers a comprehensive overview of ankle exoskeletons, including existing examples, kinematic principles, and modeling methodologies employed in the design process. Additionally, numerical characteristics are provided to validate the feasibility and efficacy of the proposed exoskeleton design.

By offering a systematic exploration of relevant topics, this paper aims to contribute valuable insights to the field of assistive technologies and advance the development of innovative solutions for enhancing human mobility and rehabilitation practices.

## 3. Materials and Methods

This section elucidates the methodology employed in developing the proposed rehabilitation device, adhering to pertinent standards. Initially, it was imperative to ascertain the requisite movement criteria examining the biomechanics of the human ankle joint, with particular emphasis on its mobility aspects. Subsequently, a device was introduced that incorporates three degrees of freedom and outlines the structure of the control system. This phase culminated in the simulation of the performance characteristics of the exoskeleton, followed by a comprehensive discussion of the obtained results.

### 3.1. Anatomy of the Ankle Joint

The ankle joint complex, far from being a mere hinge joint, exhibits a sophisticated arrangement of multi-axial motions that work synergistically to support the intricacies of human locomotion. This complexity is rooted in a comprehensive understanding of the anatomical structure and kinematics of the human foot [43]. Comprising multiple interlocking joints, the human ankle is characterized by the positioning of the talus bone at its core, flanked by the cuboid and navicular bones. The superior facet of the talus interfaces intricately with the tibia and fibula, forming the upper ankle joint (UAJ). Through the UAJ, the ankle facilitates pivotal movements such as plantarflexion and rotational dorsiflexion which are crucial for various activities ranging from walking to running [44].

The capacity of the ankle joint is further enriched by the interplay of the forefoot bones, where interconnected articulations permit nuanced inversion and eversion rotations. This intricate arrangement ensures the ankle joint’s adaptability to dynamic changes in terrain and movement requirements [45].

**Figure 1:**
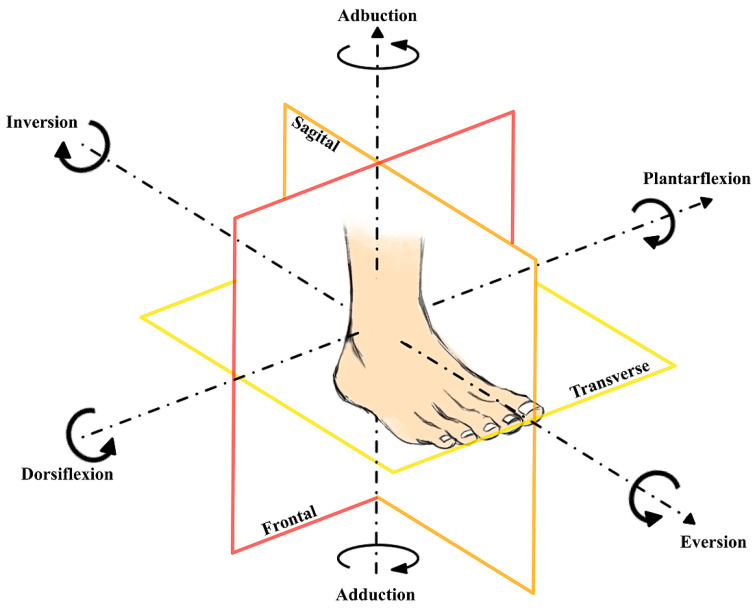
Main foot rotations around the two axes of the ankle.

Figure 1 serves as a visual aid, elucidating the complex interplay of these movements within the ankle joint.

The extensive body of literature on ankle-foot anatomy [46] underscores the significance of comprehending the underlying structural and functional nuances. This knowledge base is essential for guiding subsequent research endeavors to elucidate the intricate mechanisms governing ankle-foot movements and inform the development of robotic exoskeletons.

Range of motion (ROM) means the extent or limit at which a part of the body can move around a joint or fixed point and the set of movements that the joint can perform. ROM is assessed during evaluation or treatment in physiotherapy, and its normal values depend on the body part and individual’s characteristics. The range of motion can vary greatly among people due to geographical and cultural differences, anatomical structure, and different data collection methods. To determine the target range of movements for the ankle support device, conservative values for the ankle movement range as shown in Figure 1 and Table 1 were taken which corresponded to daily activities.

In the quest to develop robotic exoskeletons that accurately replicate natural ankle movements, a diverse array of studies [47,48,49,50,51] have been conducted. These studies explore the biomechanical principles governing ankle kinematics, paving the way for innovative designs that enhance mobility and rehabilitation outcomes for individuals with impaired ankle function.

### 3.2. The Proposed Device

Rehabilitation devices, due to their intimate interface with the human body, necessitate adherence to stringent design criteria. As the ankle joint and foot function around a fixed axis, the mechanical structure of these devices must be precisely designed to replicate the anatomical contours of the human body. This design necessity allows the device to accommodate patients of different heights, weights, and ages, making the rehabilitation process more adaptable and effective.

Beyond the considerations of safety and comfort, factors such as the normal range of motion and operational speed are of paramount importance when interfacing with a patient undergoing rehabilitation. These parameters serve as crucial benchmarks in the design and implementation of rehabilitation devices, ensuring their efficacy and alignment with therapeutic objectives.

**Figure 2:**
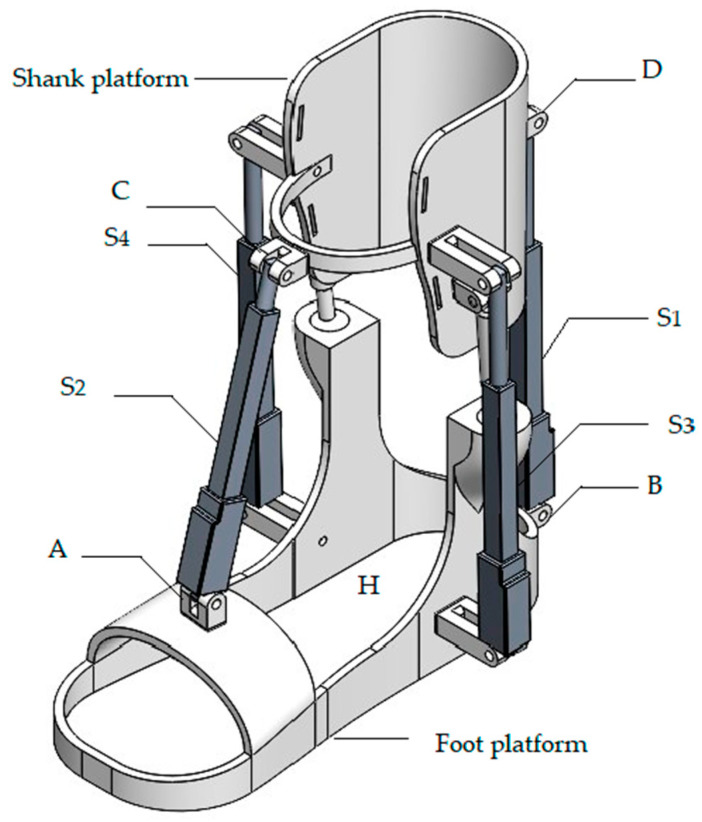
CAD model of the ankle exoskeleton components and their assembly in SolidWorks.

Figure 2 illustrates a CAD design indicating attachment points A, B, C, D for the linear actuators S1, S2 and S3, S4, with point H as the center point of the foot platform. Connections E and F on the support link the ball joint, serving as the guide between the shank and the foot platform. The calculated parameters of the CAD solution (Figure 2) are shown in Table 2.

**Figure 3:**
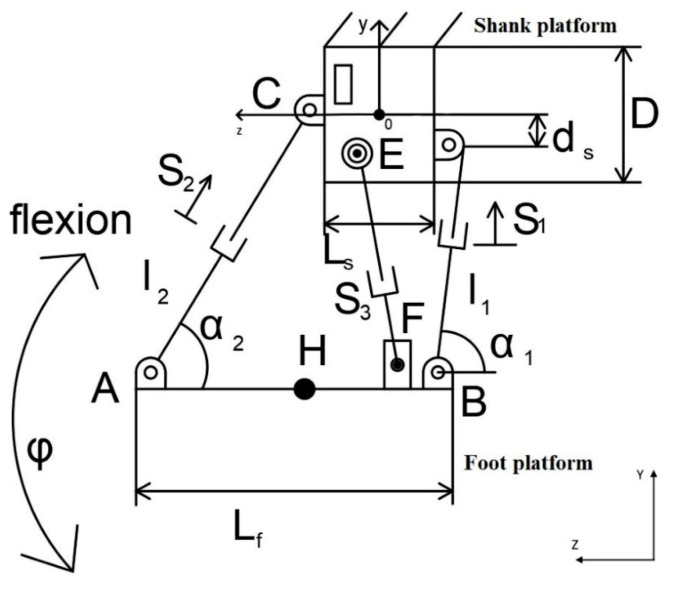
The kinematic design of the ankle exoskeleton.

The mechanism design shown in Figure 3 operates with four linear electric actuators, which are installed in parallel with spherical hinges between the lower leg and the ankle joint. The translational movement of the linear electric actuators facilitates the rotation of the ankle and the ankle joint.

According to the Somov-Malyshev formula, the number of degrees W of freedom of the mechanism for a spatial kinematic structure is determined as follows:

(1) $$W=6\;\times \;(n-1)-5\times p_{5}=6\times (8-1)-5\times 9=3$$

The application of this formula is possible if no additional conditions are imposed on the movements of the links that make up the mechanism (the axes of all rotational pairs were parallel, intersected at one point, etc.). These additional requirements change the nature of the movements of the mechanism and, accordingly, change the form of its structural formula

In a spherical mechanism, all three kinematic chains impose the same connections, and the axes of all pairs intersect at one point. In the proposed design there are three power screws and three kinematic mutual screws. These are the zero-parameter screws. To determine the number of degrees of freedom, we apply the Dobrowolski formula as follows:

(2) $$W=3\times (n-1)-2\times p_{5}-p_{4}=3\times (8-1)-2\times 9=3$$

If the last rotational pairs are replaced by spherical ones, then in this case each chain imposes one bond. The number of degrees of freedom is determined by the Somov-Malyshev formula as follows:

(3) $$W=6\times \left(n-1\right)-5\times p_{5}-4\times p_{4}-3\times p_{3}=6\times (8-1)-5\times 6-3\times 3=3$$

Figure 3 presents the kinematic design of the ankle exoskeleton along with its parameters.

Based on the kinematics of the connections in the design of the exoskeleton (shown in Figure 3), the articulation angles of the joints can be described by the following.

The relative motion of the platform attached to the foot compared to the platform attached to the calf can be expressed as the following:

(4) R   AB=Rz(α)Ry(φ)Rx(θ),

where φ is the dorsiflexion/plantarflexion, θ is the inversion/eversion, and α is the abduction/adduction.

When examining relative motion, it is important to consider the following three aspects: (a) ensuring constant tension in all actuators, (b) utilizing spherical hinges at the attachment points of the actuators to the platform, and (c) treating the actuators as prismatic joints with minimal axial deformation.

The points on which the drives on the platform attached to the ankle can be generically labeled $A_{a_{i}}={\left(a_{ix},a_{iy},a_{iz}\right)}^{T}$, and the points on the platform attached to the foot can be labeled $B_{a_{i}}={\left(b_{ix}{,b}_{iy}{,b}_{iz}\right)}^{T}$.

Rehabilitation exercises require the execution of homogeneous, slow, and controlled movements so that the patient feels the least stress or pain. Since these exercises are performed at a limited speed, inertial effects and movement dynamics can be ignored in the analyses. Therefore, static analysis can be used to evaluate the effectiveness of the robot.

The voltage in each drive is defined as $T_{i}=-T_{i}p_{i}$ as the product of the unit vector and its intensity, and the vector T here ${\left(T_{1}T_{2}T_{3}T_{4}\right)}^{T}$:

(5) $$P^{T}\cdot T-F_{R}=F_{ext};$$

and

(6) $$Q^{T}\cdot T-M_{R}=M_{ext},$$

where $P^{T}=\left[p_{1}p_{2}p_{3}p_{4}\right]$ and $Q^{T}=\left[b_{1}\times p_{1}\ldots b_{4}\times p_{4}\right]$.

As previously stated, due to the spherical nature of the kinematic representation of the ankle joint and the unrestricted range of rotational movement, the reaction moment $M_{R}\;$is a zero vector. Consequently, the complete equilibrium equation can be formulated as follows:

(7) $$\left[P^{T}-I_{3}\;Q^{T}\;0_{3}\;\right]\left(T\;F_{R}\;\right)=\left(F_{ext}\;M_{ext}\;\right),$$

The challenge in robot operation can be characterized by solving for torque based on the movement of the ankle joint.

**Figure 4:**
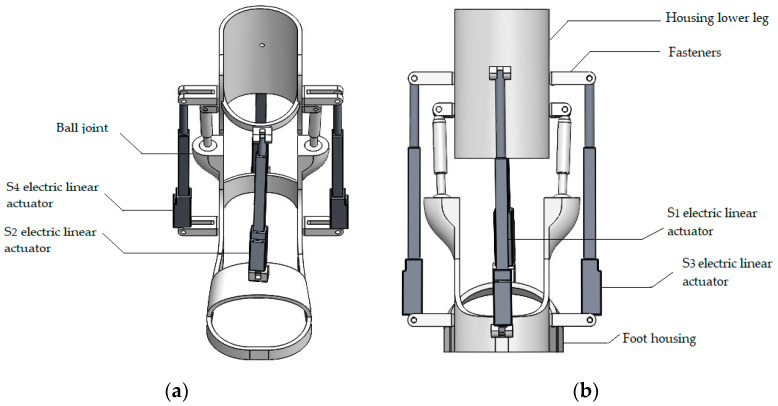
Conceptual design of the ankle exoskeleton. (**a**) General front view. (**b**) General view in the back.

According to the conceptual model of the ankle exoskeleton design shown in Figure 4, the electric linear actuator should integrate a system that coordinates the movement of the two interconnected skeletons thereby moving the ankle along with the exoskeleton. When assessing relative motion, it is crucial to consider the following three conditions: (a) ensuring constant tension in all actuators, (b) utilizing spherical hinges at the attachment points of the actuators to the platform, and (c) treating the actuators as prismatic joints with minimal axial deformation.

### 3.3. Performance Characteristics Simulation

A 3D modeling and simulation analysis was conducted within a virtual framework employing the SolidWorks Simulation Professional software alongside the Motion Simulation adjunct. By applying an electric linear actuator input via SolidWorks Simulation, dynamic articulation of the ankle joint was achieved.

**Figure 5:**
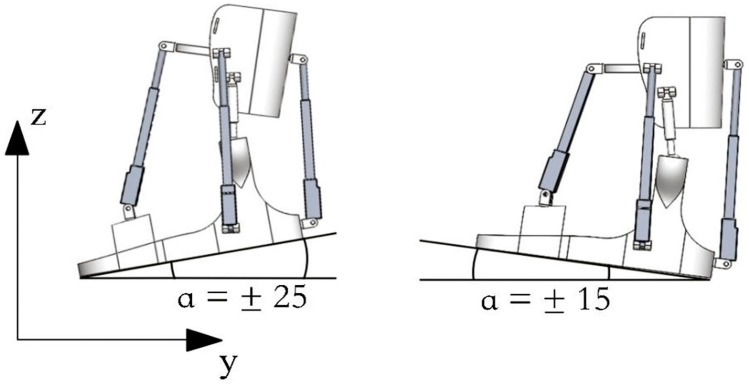
A snapshot showcasing simulated assisted movements in dorsiflexion and plantarflexion.

Furthermore, Figure 5 showcases the dorsal and plantar flexion movements of the ankle joint in a neutral position, providing a visual representation of simulated assisted movements facilitated by SolidWorks Simulation. Dorsal flexion exhibits a range of motion up to 20 degrees, with a simulated bending depicted in 15 degrees. On the other hand, plantar flexion demonstrates a range of motion spanning from 40 to 50 degrees, with a simulated bending capability of up to 20 degrees.

**Figure 6:**
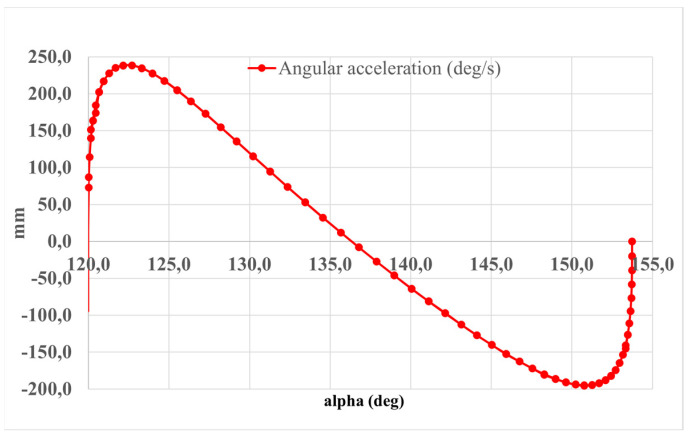
The computed results of the simulated motion portrayed in Figure 5 presented in terms of the α angle of the foot platform.

Figure 6 depicts a graph illustrating the relationship between angular acceleration and foot angle (α). The graphical representation delineates notable features of the acceleration profile. A prominent peak is observed at the upper extremity of the motion, reaching a magnitude of 240 deg/s^2^. Similarly, another significant peak, measuring 150 deg/s^2^, is discernible near the lower limit of the motion trajectory. These distinctive peaks signify critical instances of rapid angular acceleration within the system under investigation.

**Figure 7:**
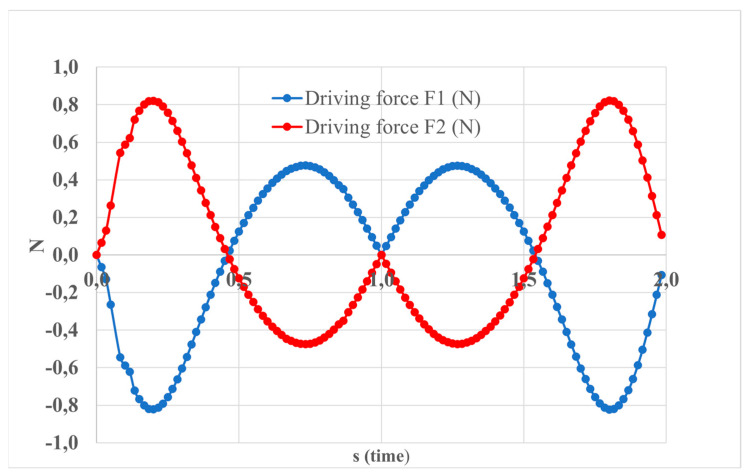
The computed results of the simulated motion depicted in Figure 5 expressed in terms of the driving force exerted by the linear actuators.

Figure 7 depicts the driving force applied by the linear actuators, with F1 representing the force from the actuator on the front side of the leg, and F2 indicating the force from the actuator on the back side of the leg. Computational analysis shows that both forces, F1 and F2, achieve a magnitude of 0.8 N.

**Figure 8:**
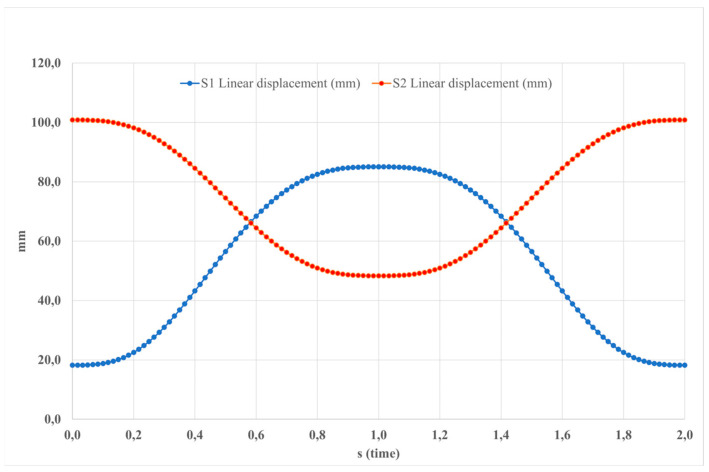
The input data for the simulated motion depicted in Figure 5 pertains to the displacement of linear actuators.

Figure 8 illustrates the components of linear displacement of the platform. The graph displays peaks in all directions, with maximum values reaching 100 deg/s^2^ for the x-component and less than 85 deg/s^2^ for the other components.

**Figure 9:**
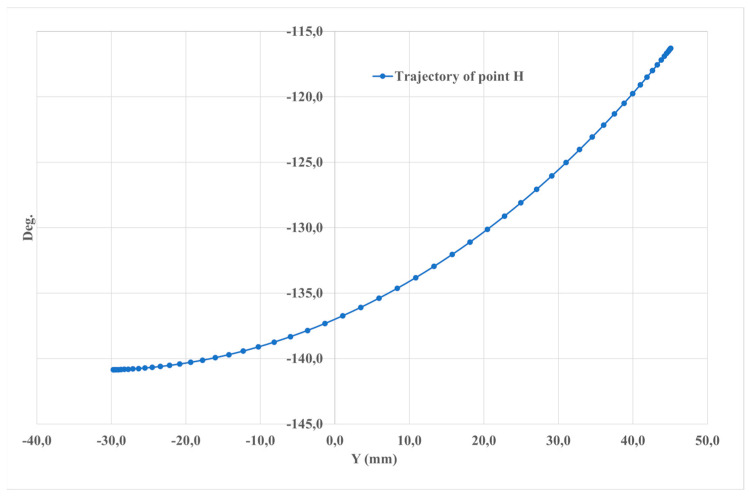
The computed results of the simulated motion illustrated in Figure 4 described relative to the trajectory of point H on the foot platform.

Figure 9 shows the path traced by point H, positioned at the center of the platform. The displacement of this point along the z-axis is 25.5 mm, and along the y-axis, it reaches 74.6 mm, corresponding to an angle of +/− 8 degrees.

**Figure 10:**
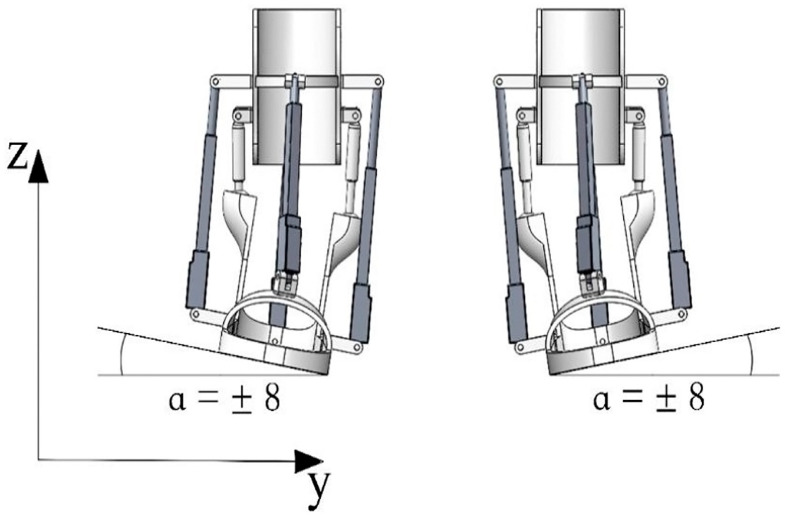
A snapshot depicting simulated assisted motions in abduction and adduction.

Figure 10 presents a snapshot depicting simulated assisted motions in abduction and adduction.

**Figure 11:**
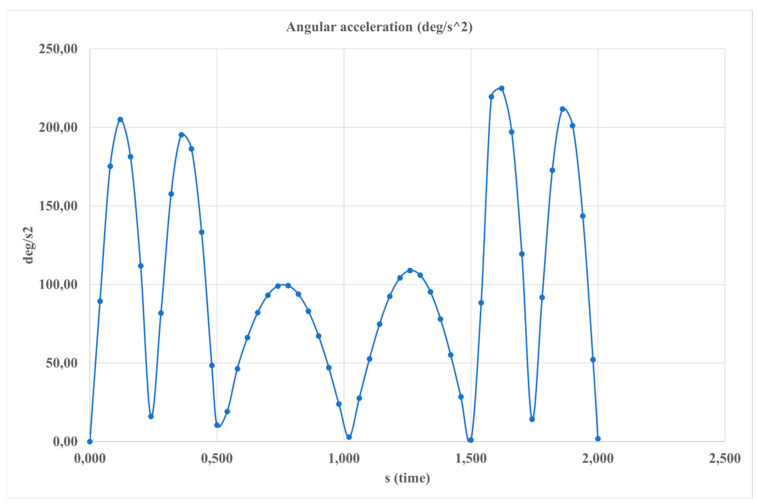
The computed results of the simulated motion depicted in Figure 4 expressed in terms of the angle of the foot platform.

Figure 11 illustrates the angular acceleration with respect to the angle. The highest acceleration value, peaking at 23 deg/s^2^, occurs near the top position. Angular acceleration quantifies the rate of change in both the magnitude and direction of angular velocity as the ankle joint moves with the exoskeleton.

**Figure 12:**
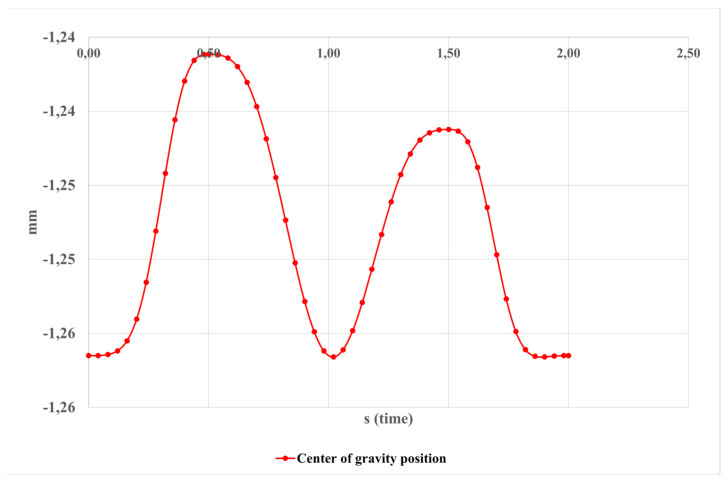
The input data for the simulated motion depicted in Figure 10 including the center of gravity position.

Figure 12 presents the computed outcomes regarding the components of the platform’s center of gravity position. The displacement of this point along the y-axis is measured as −1.23 mm. In this study, the center of gravity signifies the stability of equilibrium positions of bodies and continuous media under the influence of gravity. This concept is particularly relevant in the analysis of material resistance, where it is utilized in conjunction with the Vereshchagin rule.

**Figure 13:**
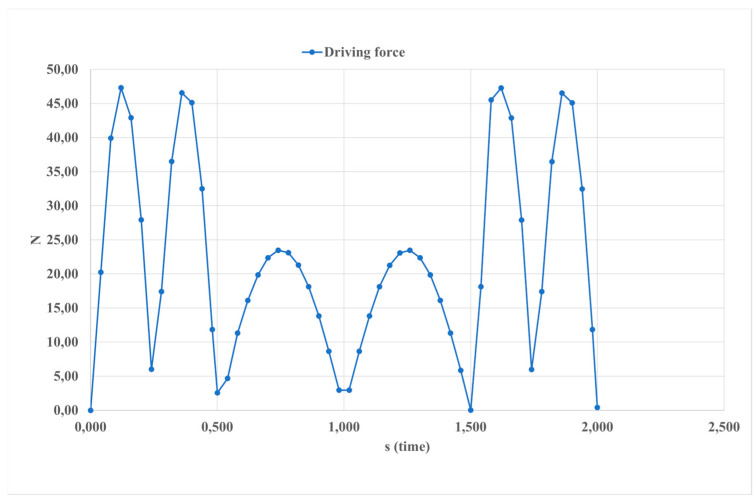
The calculated results of the motion simulation, as shown in Figure 10, presented in terms of the driving force.

The driving force depicted in Figure 13 represents an external force exerted on the system to sustain motion. It symbolizes the force generated by linear actuators to induce movement in the ankle joint, exhibiting a maximum value of 45 Newton and a minimum value of 25 Newton in the motion simulation. Understanding this force is essential for comprehending the dynamics and mechanics of the system under investigation.

**Figure 14:**
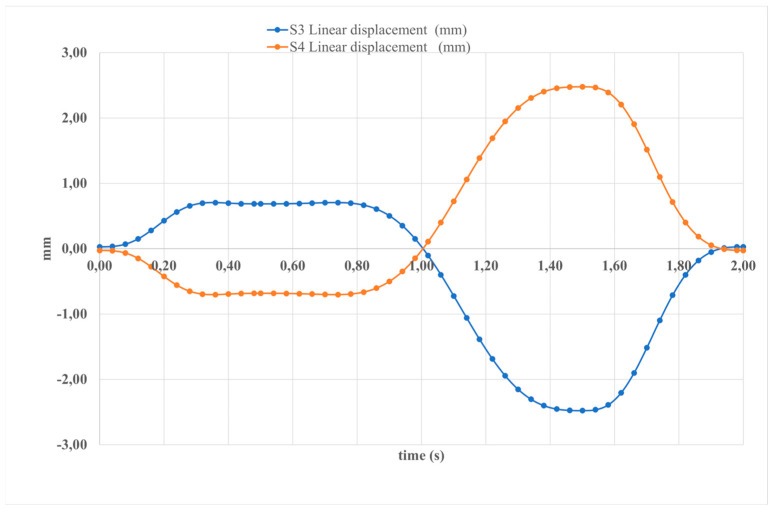
The computed results of the simulated motion depicted in Figure 10 presented in terms of the displacement of linear actuators.

Figure 14 shows the components of linear displacement of the linear actuators. The figure indicates that movements in all directions reach peaks, with maximum values of 30 deg/s^2^ for the y-component and less than 25 deg/s^2^ for the other components.

**Figure 15:**
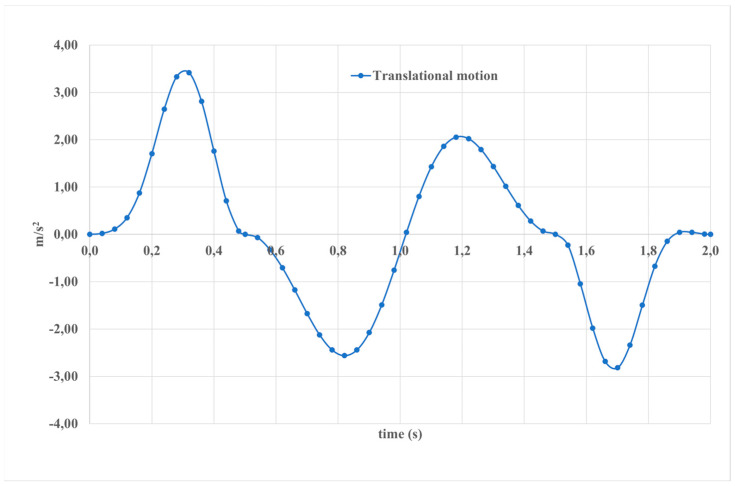
The calculated results of the simulated motion depicted in Figure 10 are presented in terms of translational motion.

Figure 15 illustrates translational motion, also known as object motion, wherein each point of the object follows a parallel trajectory along straight lines. This stands in contrast to rotational motion, where an object rotates around a fixed axis. In the depicted scenario, over a 2-s interval, the upper part demonstrates a displacement of 3.5 m/s^2^, while the lower part exhibits a displacement of 2.8 m/s^2^. Understanding translational motion is paramount in the context of an exoskeleton as it provides the foundation for analyzing the spatial movement, velocity, and acceleration of objects within the system.

**Figure 16:**
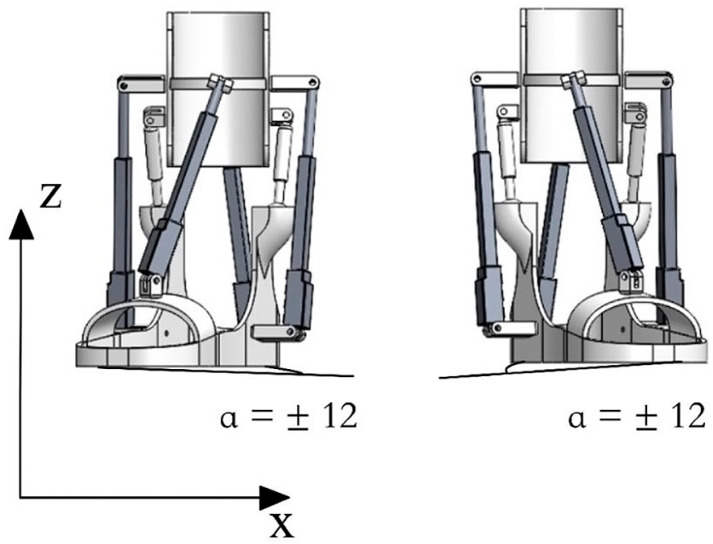
A snapshot illustrating simulated assisted motions in inversion and eversion.

The provided illustrations depict the simulated motions of both inversion and eversion achieved using SolidWorks Simulation. The range of motion for inversion spans from 14.5 to 22.0 degrees, reaching 12 degrees in the depicted simulation. For eversion, the movement ranges from 10.0 to 17.0 degrees, with a simulated bending capability of up to 12 degrees. Figure 16 presents a snapshot illustrating simulated assisted motions in inversion and eversion.

**Figure 17:**
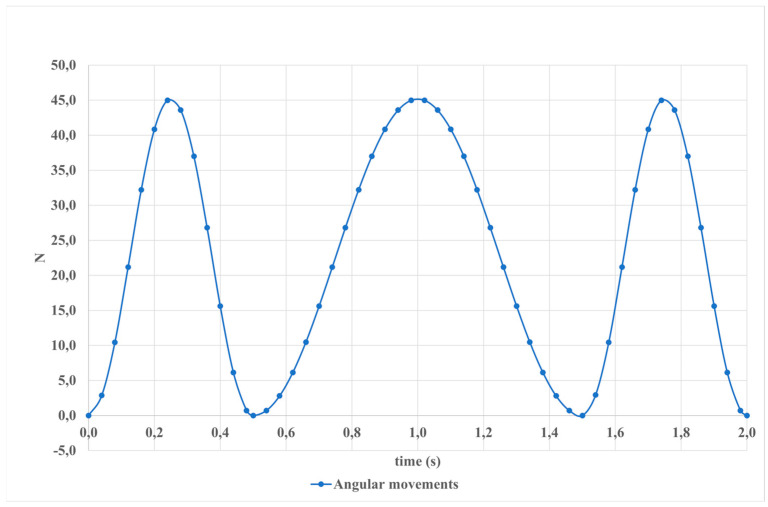
The results of motion modeling utilizing linear actuators as depicted in Figure 16, showcasing angular movements.

Angular movements arise from changes in the angle between the bones that make up a joint, demonstrating the kinematic dynamics of the system. Figure 17 visually represents these angular movements. Within a 2-s interval, a torque of 45 Newton-seconds is observed, underscoring the dynamic forces involved in angular motion. Understanding such angular kinetics is fundamental in biomechanical analyses, offering insights into joint function and movement patterns.

**Figure 18:**
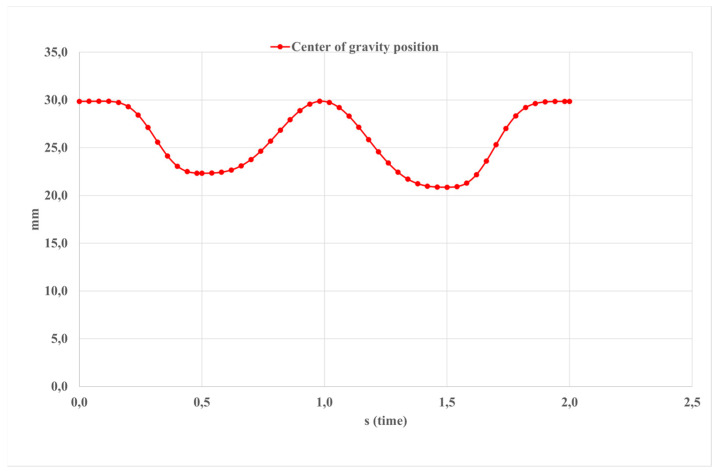
Simulation results for the motion in Figure 16 by the center of gravity.

Figure 18 presents the calculated outcomes of the platform’s center of gravity components. The displacement of this point along the z-axis measures 30 mm. In this study, the center of gravity signifies the stability of equilibrium positions for bodies and continuous media under the influence of gravity, particularly in applications such as material resistance where the Vereshchagin rule is applied.

The center of gravity (COG) of the human body represents a theoretical point where the force of gravity is considered to act. It is seen as the focal point around which the combined mass of the body is concentrated. However, due to the dynamic nature of human movement and variations in body proportions, the exact location of the center of gravity constantly shifts with changes in body position and limb arrangement.

**Figure 19:**
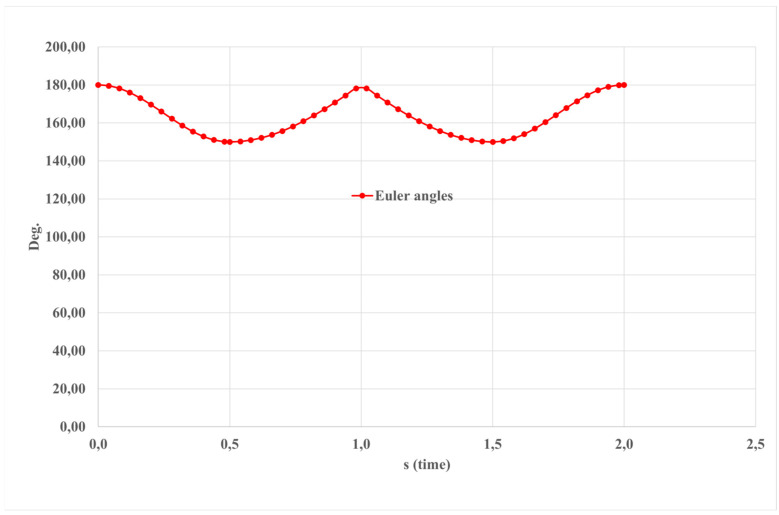
The input data for the simulated motion depicted in Figure 16 is expressed in terms of Euler angles.

The Euler angles depicted in Figure 19 elucidate a sequential amalgamation of passive rotations around the axes of a rotating coordinate system. This representation facilitates a precise description and analysis of the complex rotational movements, thereby enhancing our comprehension of dynamic systems and their behavior.

**Figure 20:**
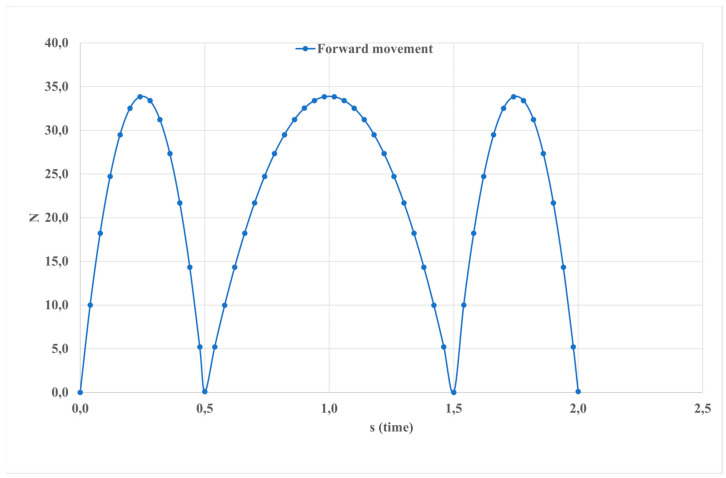
Forward movement.

Forward motion pertains to the displacement occurring along the orientation of an object or in alignment with the intended path of motion. This concept holds significance in biomechanics, where it delineates the progression of anatomical structures during locomotion. The accompanying Figure 20 illustrates that within a 2-s timeframe, a peak force of 35 Newtons is exerted, highlighting the biomechanical dynamics involved in ankle movement. Understanding such forces is instrumental in deciphering the intricacies of human locomotion and optimizing performance in various physical activities.

## 4. Results

### 4.1. Assembly of an Ankle Exoskeleton Prototype

**Figure 21:**
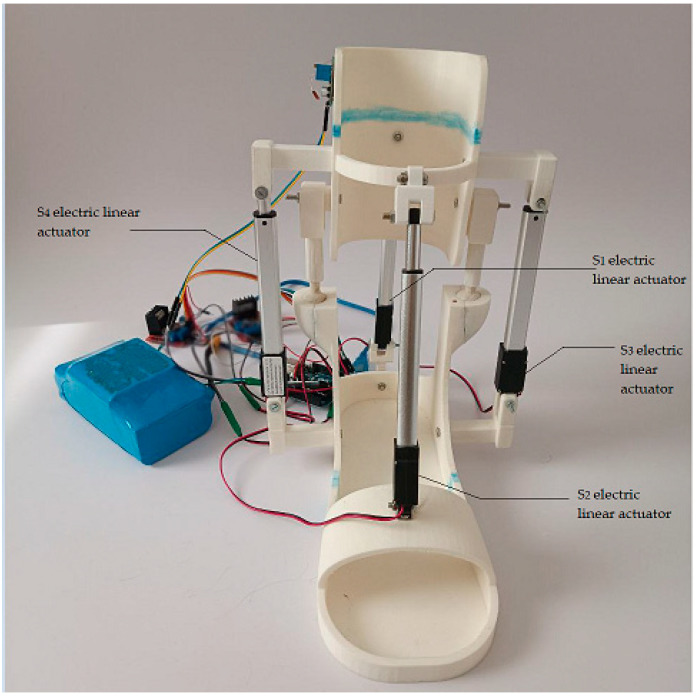
General view of the exoskeleton prototype.

The design results represent a novel approach to addressing mobility problems associated with ankle dysfunction by developing a prototype of a low-cost exoskeleton. The exoskeleton prototype is assembled to validate the developed concept in a practical application virtually. The primary and support structures of the exoskeleton prototype are fabricated using a Creality Ender-3 V2 3D printer. The base of the ankle exoskeleton body is made of PLA, which contributes to the structural integrity and strength of the exoskeleton’s outer shell. This is important because the prototype system must withstand the mechanical forces and loads generated by ankle joint movement while ensuring stable device performance during rehabilitation. When designing the exoskeleton body, ease of assembly was prioritized to provide comfort and convenience for clinicians and patients. Figure 21 presents the assembled prototype of the exoskeleton, combining the mechanical and electronic components. The weight of the prototype for each foot individually is 2.05 kg.

The prototype features specially placed attachment points that facilitate the smooth integration of various components, including actuators, sensors, and fastening mechanisms that secure the exoskeleton to the user’s lower limb. Ergonomics plays a crucial role in the design of the body. Ergonomic considerations include reducing pressure points and optimizing fit and weight distribution to ensure user comfort. The appearance of the PLA exoskeleton body has been carefully designed to be aesthetically pleasing.

In its design, customization, durability, weight optimization, ergonomic considerations, safety features, and ease of assembly were prioritized. All these attributes combine to ensure the effectiveness, safety, and comfort of the exoskeleton user in various rehabilitation and mobility applications. Table 3 summarizes the main device performance parameters used in the assembly of the exoskeleton prototype.

### 4.2. Solution for Control Design Unit

The control system of the robotic exoskeleton, driven by a linear electric actuator, incorporates position and power control units for the platform. This setup allows for generating torque for the robot’s motion. Active training includes resistance mode and combination mode. In resistance mode, the motor applies resistance, necessitating extra effort from the user to move the platform. This mode also generates a counterforce to enhance joint strength. Conversely, in combination mode, the user’s movement creates torque opposing the applied force.

In contrast, passive assist mode relies solely on the motor for all leg movements on the platform, eliminating the need for user muscular activity. It encompasses voluntary and compulsory passive exercises, with the latter extending joint movement beyond the user’s active range, necessitating motor assistance. This study focuses on the limited motion range within which the robot operates.

The sensor components in the system are divided into two primary groups. Control sensors, including IMU sensors, EMG sensors, and voltage sensors which deliver essential data such as angular position, platform position, real-time cable length, and voltage readings. Force sensors gauge user-applied force and discern intended platform maneuvers. Additionally, a blood pressure transducer monitors ankle joint pressure, ensuring patient safety during rehabilitation.

The ankle exoskeleton’s control scheme, depicted in the provided Figure 4 functions as follows:

- Inputs to MCU (microcontroller unit): received from various sources including a PC, force-sensitive resistors (FSR), an EMG sensor, and an IMU sensor;
- MCU outputs: processes inputs to control additional devices for enhanced functionality and a DC motor driver, which regulates the DC motor;
- Ankle joint manipulation: the DC motor manipulates the ankle joint, enabling movement and support by the exoskeleton.

**Figure 22:**
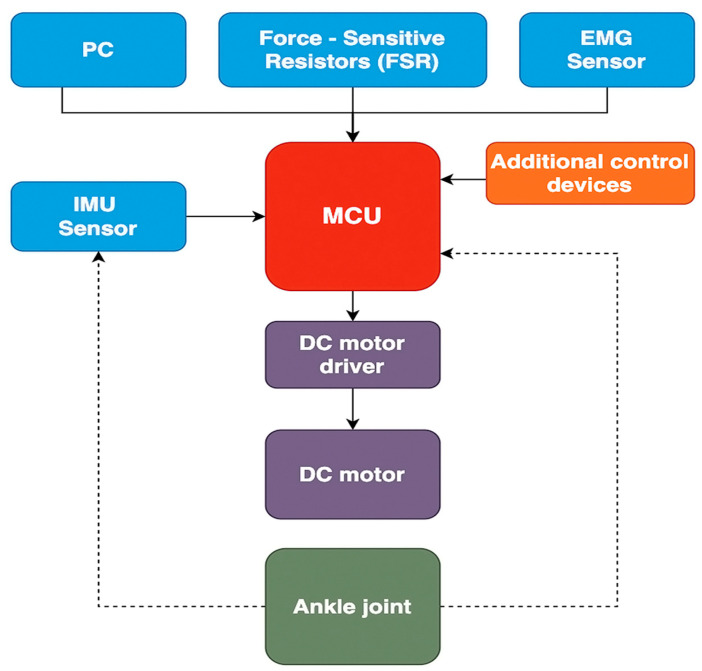
A scheme for the control design for the ankle joint exoskeleton.

The following flowchart (Figure 22) illustrates the integration of hardware and software components to precisely control the ankle exoskeleton’s movements, utilizing sensors for real-time feedback and adjustments to ensure safe and effective operation.

All the aforementioned components of the prototype control system are interfaced using an Arduino microcontroller, which serves as the central processor of the system. The Arduino receives signals from the sensors and converts them into commands for the actuators, which regulate the linear actuators. In addition, the microcontroller performs other functions such as data logging, processing sensor data, and communicating with other devices.

### 4.3. Experimental Study and Functional Testing of the Prototype

**Figure 23:**
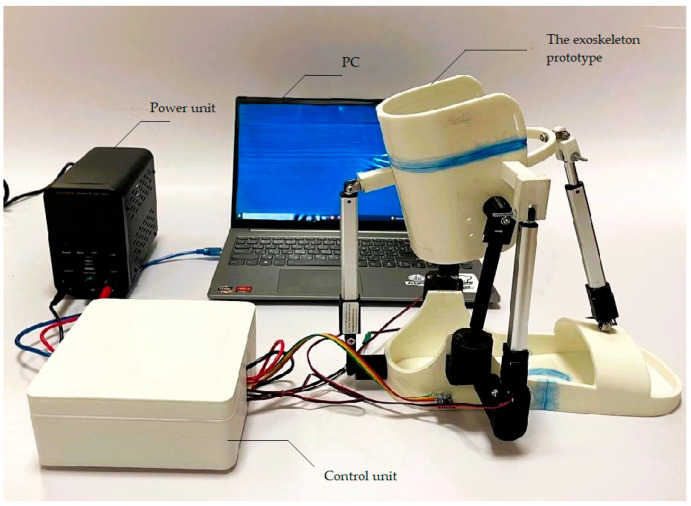
Testing layout with the main components of the ankle exoskeleton.

The testing layout with the main components of the ankle exoskeleton shown in Figure 23 consists of a battery power supply (Power unit), ankle joint platform (exoskeleton prototype), drives, ball joint, microcontroller, drivers (control unit system), and PC.

**Figure 24:**
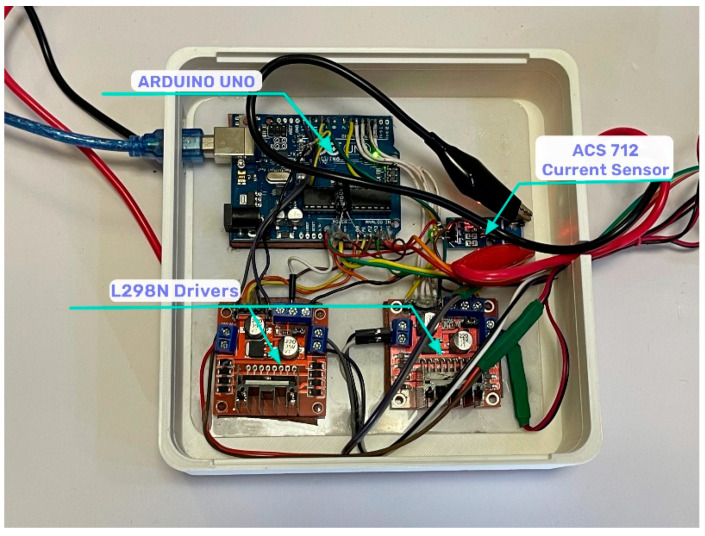
The control system unit.

The control system unit, shown in Figure 24, consists of sensors that provide essential feedback to the control system, allowing it to adapt the signals applied to the electric linear actuators.

Actuonix linear actuators contain a built-in DC motor that can be controlled with the L298N driver. The L298N driver is used to control actuators as a direct current motor and contains two H-bridge channels, which allows the control of two motors simultaneously. The actuator receives a rated voltage of 12 V, corresponding to its specification.

To ensure that the required specifications were met after the design and assembly of the exoskeleton prototype, functional testing of the device was performed to evaluate the performance of the four linear electric actuators. The results of the functional testing evaluated the exoskeleton prototype’s functionality, usability, and safety under various loads and conditions.

Modeling the dynamics of an ankle rehabilitation device considering three basic motions is a challenging task due to the nonlinear nature of the overall system; in this case, the main objective of the mechatronic system of the exoskeleton prototype is to control the devices based on simplified models, thus increasing their robustness to external influences. With this in mind, the separate dynamics required to maintain the basic movements of the ankle rehabilitation device were initially investigated. This is because, in passive rehabilitation, specific exercises are first performed to ensure stability and functionality of the ankle joint.

**Figure 25:**
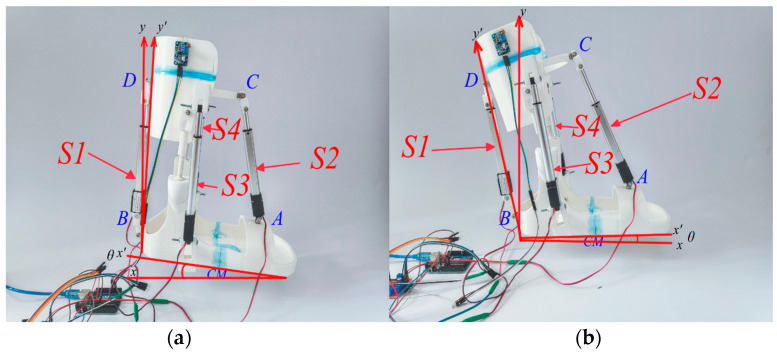
Experiment of functional testing of the ankle joint exoskeleton during linear motion in dorsiflexion and plantarflexion movements. (**a**) Dorsiflexion. (**b**) Plantarflexion.

Figure 25 shows a device used for experimental functional testing of the ankle exoskeleton during movements in the dorsal and plantar flexion. The test evaluates the effectiveness of the exoskeleton and the operation of the actuators.

All dorsiflexion–plantarflexion movements are recorded by the IMU BMI160 sensor.

**Figure 26:**
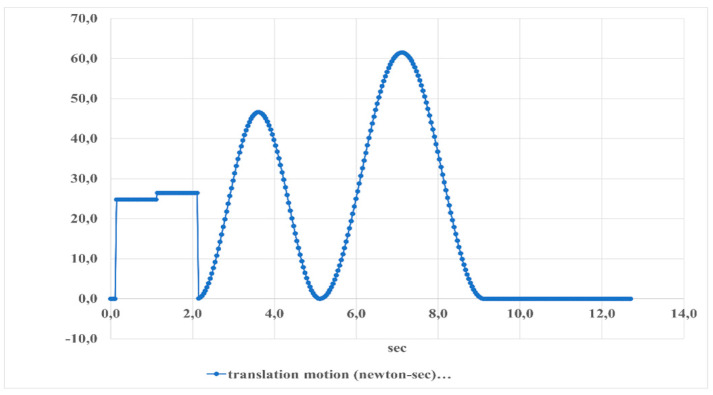
The results of the translation motion of the foot platform during dorsiflexion–plantarflexion movements are shown in Figure 25.

Figure 26 shows the translation motion of the foot platform during dorsiflexion–plantarflexion movements over time. The motion fluctuates between approximately −10 and 70 Newton-seconds, indicating the force exerted in different directions. This periodic pattern repeats approximately every 14 s, reflecting the cyclical nature of these movements.

**Figure 27:**
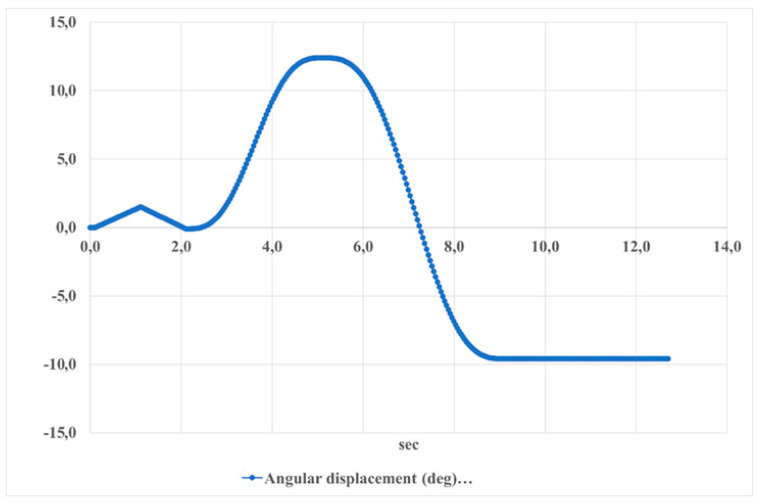
The results of the angular displacement of the foot platform during dorsiflexion–plantarflexion movements are shown in Figure 25.

Figure 27 presents the angular displacement of the foot platform during dorsiflexion–plantarflexion movements over time. The displacement ranges from approximately 10 to −10 degrees, indicating the range of motion of the ankle joint. This periodic pattern reflects the regular cycle of these movements, which is important for understanding the dynamics of foot motion.

**Figure 28:**
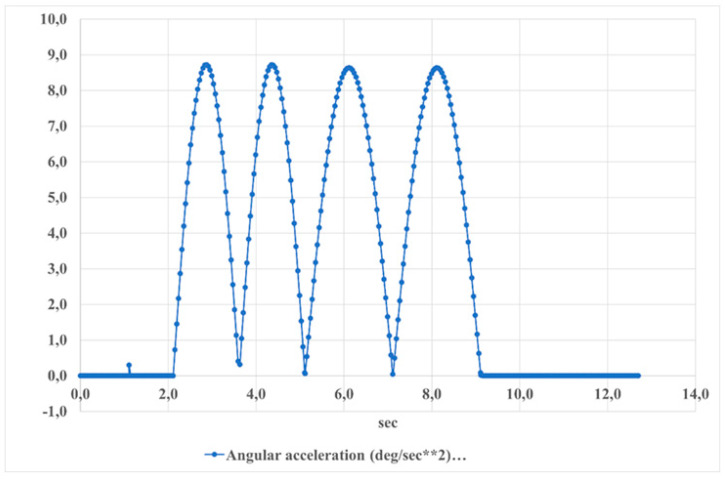
Angular acceleration of the foot platform during dorsiflexion–plantarflexion movements is shown in Figure 25.

Figure 28 shows the angular acceleration of the foot platform during dorsiflexion–plantarflexion movements over time. The angular acceleration ranges from approximately −1 to 9 deg/s^2^. This pattern repeats approximately every 2 s, indicating a regular cycle of movement.

**Figure 29:**
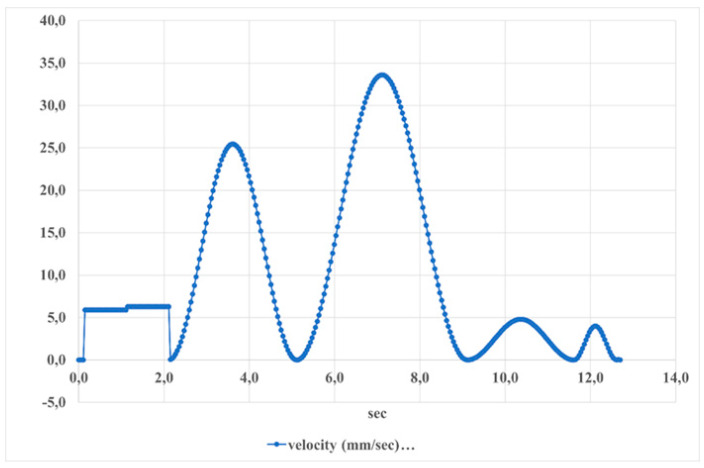
The speed of the foot platform during dorsiflexion–plantarflexion movements is shown in Figure 25.

Figure 29 shows the velocity of the foot platform during dorsiflexion movements. The velocity ranges from approximately 35 mm/s to −5 mm/s, indicating a periodicity of the movement. This pattern repeats approximately every 4 s, reflecting the cyclical nature of the foot platform velocity during these movements.

**Figure 30:**
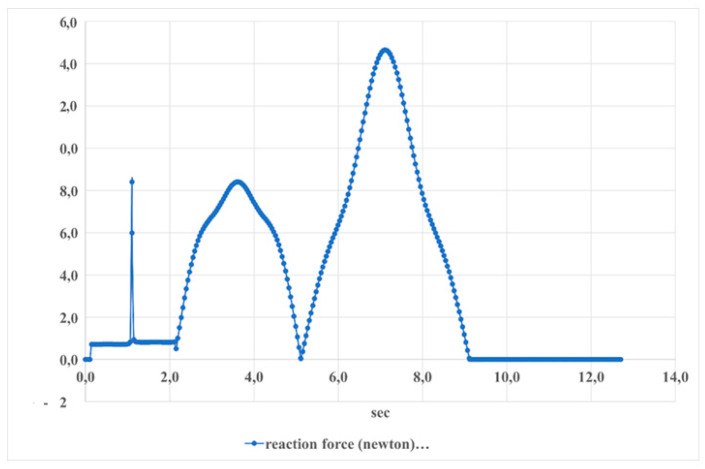
Reaction force of the actuator (S1) when moving the dorsiflexion–plantarflexion as shown in Figure 25.

Figure 30 presents the reaction force of the S1 actuator during dorsiflexion–plantarflexion movements over time. The force starts at 0 Newtons, peaks at approximately 20 Newtons at 2 s, returns to 0, then increases again to just over 50 Newtons at about 8 s before returning back to 0. This indicates a change in the force exerted by the actuator during these movements.

This demonstrates that the result values are approaching the maximum actuator force value of 50 N.

**Figure 31:**
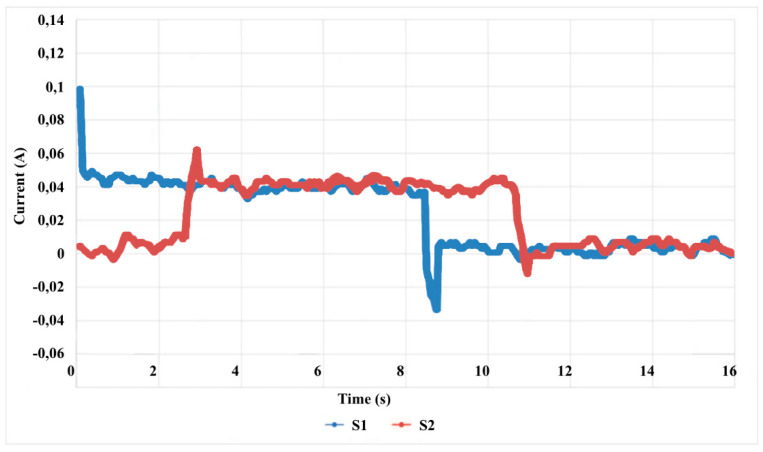
Current values in actuators S1 and S2 over time.

In functional testing, the actuators have current values of: S1—0.1 A and S2—0.06 A. This is due to the fact that the drives move the platform back and forth alternately, repeating the movements of the input and output as shown in Figure 31, where the current values are given in drives S1 and S2.

Actuonix, Miniature Linear Actuators L16 [52] used in the exoskeleton has a compact and lightweight design, making it ideal for integration into the exoskeleton without significant increases in volume or weight.

In this pilot study, the functional testing results demonstrate the ability of the exoskeleton prototype to generate controlled linear and angular movements with significant accelerations in a short period of time. This highlights its potential to improve mobility, stability, and support during dynamic activities.

The functional testing results obtained during the experiment play a key role in improving the exoskeleton design and optimizing its real-world performance.

## 5. Conclusions

In this study, a virtual model of the proposed exoskeleton was developed and simulated using SolidWorks Motion Simulation software. Additionally, a low-cost prototype of the exoskeleton for ankle rehabilitation was created and tested. The prototype represents an innovative approach to address mobility problems associated with ankle joint dysfunction. The study also focused on the complex anatomy and kinematics of the ankle joint, which was considered when developing a comprehensive computer-aided design system model that allows simulation analysis to evaluate the viability and performance of the prototype design. It has four linear electric actuators that provide three bare ankle joint movements: dorsal flexion and plantar flexion, adduction, abduction, and inversion and eversion. The presence of 3DOFs in the prototype is able to mimic ankle joint motion, allowing for a smoother and more natural walking motion. This helps to speed up the recovery process for patients with disabilities. The use of modern 3D printing technologies and the choice of available materials to assemble the exoskeleton prototype increased its cost-effectiveness. This reduces initial production costs and facilitates maintenance and repair of the device during operation, an essential key factor for long-term operation and maintenance of its performance. The use of modern sensors and electronics allows the exoskeleton prototype to accurately track movements and adapt the functionality of the device to the needs of the user, making it attractive to a wide range of users.

The exoskeleton’s control system, which includes positional and force control units, enables the generation of the required torque for the robot’s movements. The use of sensors to monitor angular position and force ensures accurate and safe operation of the device. The results of the functional testing showed that each of the linear actuators operated correctly. This phase included testing the motor, gearbox, and other mechanical components, as well as the control electronics and software, which demonstrated their efficiency and the functionality of the system.

Once the design phase is complete, the prototype is assembled to validate the concept under practical conditions. Future research will focus on conducting experimental studies to evaluate the functionality and performance of the exoskeleton in real-world environments. After functional testing in real-world conditions is completed, the next key step is to perform load testing, assessing speed under various loads and conditions, and testing the durability of all components of the exoskeleton system. Safety testing will also be conducted, which will include checking for potential risks, such as electrical shock or mechanical failures, and ensuring that the actuators meet all necessary standards and safety requirements.

This integrative approach to development and testing can significantly improve rehabilitation methods and the quality of life for individuals with ankle mobility disorders. Future research will focus on conducting experimental studies to evaluate the functionality and performance of the exoskeleton in real-world conditions. This integrative approach to design and testing has the potential to significantly improve rehabilitation methods and the quality of life for people with ankle mobility impairments.

Future endeavors will focus on conducting experimental characterization studies, providing invaluable insights into the exoskeleton’s functionality and performance under real-world conditions. This iterative approach, combining simulation-based design refinement with practical validation through prototyping, holds immense promise in the development of advanced assistive technologies.

## Figures and Tables

**Table 1:** Normal human ROM.

Motion Direction	ROM (Degree)
Dorsiflexion	20.3–29.8
Plantarflexion	37.6–45.8
Inversion	14.5–22.0
Eversion	10.0–17.0
Abduction	15.4–25.9
Adduction	22.0–36.0

**Table 2:** Design and operation parameters of a CAD solution in Figure 2.

Size	Shank Platform (mm)	Foot Platform (mm)	S1 (mm)	S2 (mm)	S3 (mm)	S4 (mm)
	200	265	243	226	181	93

**Table 3:** Parameters of the main components of the prototype.

Component	Commercial Name	Voltage	Mass	Max Force/Torque	Speed
Arduino board	Mega 2560 23	7–12 V	37 g	—	—
Linear actuator	L16-100-63-12-P 19	12 V	74 g	100 N	20 mm/s
Servomotor	MG996R 20	4.8–7.2 V	55 g	150 N-cm	461.5 deg/s
IMU	BMI16025	3–5 V	2 g	—	—
Force sensor	Sparkfun Resistive sensor	—	50 g	—	—

## Data Availability

The data presented in this study are available on request from the corresponding author.
